# The Single, the Whole, and the Pooled: Implications of Tissue Choice for Stable Isotope Analysis

**DOI:** 10.1002/jez.70122

**Published:** 2026-08-03

**Authors:** Paride Balzani, Nadège Belouard, Lukáš Veselý, Antonín Kouba

**Affiliations:** ^1^ Faculty of Fisheries and Protection of Waters, South Bohemian Research Center of Aquaculture and Biodiversity of Hydrocenoses University of South Bohemia in České Budějovice Vodňany Czech Republic; ^2^ GEOMAR Helmholtz Centre for Ocean Research Kiel Kiel Germany; ^3^ Université de Rennes, CNRS, ECOBIO (Ecosystèmes, Biodiversité, Évolution) Rennes France

**Keywords:** food webs, mixing models, niche width, stable isotopes, tissue, trophic position

## Abstract

In the exciting era of non‐invasive tissue sampling and data reuse via open access databases, it is increasingly challenging to ensure comparability of sample types for stable isotope analysis in ecology. According to the theoretical predictions about the changes in the stable isotope values between prey and consumers and tissue turnover rates, the ecological question alone should dictate the choice of the sample type used (single tissue, whole body, or pooled samples). However, the sample types have also been constrained by the body size, availability, or legal status of the study species, and stable isotope data sets are routinely obtained from a variety of sample types. While there is a general agreement that this practice may bias the ecological conclusions of stable isotope studies, the risks incurred have seldom been reviewed explicitly, let alone quantified. With both ethical aspects and open science opportunities arising in the field, the issues around sample types in stable isotope ecology must be tackled explicitly. We illustrate how comparing stable isotope values from different sample types affects niche analyses and diet reconstructions, and detail the consequences of pooling samples on stable isotope variance. We call for (1) a thorough consideration of the adequation between sample types and ecological questions, including in data harvesting from global databases, and (2) for the development of methodological studies around sample type issues and that of a systematic framework that incorporates the known variations between sample types in statistical analyses.

## Summary

Sample type influences stable isotope values, niche metrics and mixing models. We identify knowledge gaps and propose methodological priorities for future research.

## 1 Introduction

Stable isotope analysis (SIA) is a well‐established method to investigate trophic relationships, including assigning trophic levels (Post 2002) and determining the diet of a species (Nielsen et al. 2018), quantifying ecological niches (Bearhop et al. 2004; Newsome et al. 2007), and reconstructing entire food webs (Layman et al. 2012). Compared with traditional approaches such as stomach content analysis, SIA offers a longer‐term and time‐mediated insight on the trophic ecology of species, with the temporal window of information depending on the turnover rates of the tissues analyzed (Boecklen et al. 2011). SIA is based on predictable changes in the isotopic values of consumers with respect to those of their prey (trophic discrimination factors, TDFs; DeNiro and Epstein 1978; Fry 2006). Following this principle, mixing models are used to determine the proportion of each prey in the diet of a consumer, based on the topology (i.e., proximity) of their respective isotopic values (Parnell et al. 2013). These models rely on certain assumptions, such as that the consumer is in steady state with its diet (i.e., its tissues' isotopic composition completely reflects its diet; Boecklen et al. 2011) and that we know the TDFs to be applied (which nonetheless vary with the taxon, ecosystem, temperature, and so forth; Stephens et al. 2022; Veselý et al. 2024; Kuklina et al. 2026). However, the sample types that are routinely used in SIA studies are often dictated by logistical constraints that compete with theoretical requirements, such as the body size and legal status of the taxa of interest, resulting in multiple sample types used within studies, from specific tissues to bulk specimens. There is a general agreement that discrepancies in sample types may affect results and data interpretation, but the biases introduced have seldom been reviewed explicitly, let alone quantified. With the recent development of FAIR repositories for stable isotope data (e.g., IsoBank, Shipley et al. 2024; IsoFresh, Boulêtreau et al. 2025), the issues around mixing sample types in stable isotope ecology must be tackled explicitly to ensure comparability between different studies and avoid both too permissive and too conservative meta‐analysis approaches. Here, we aim to ignite a discussion, which we structure around three major avenues based on a simple illustrative example, and call for studies quantifying the biases introduced by different sample types.

## 2 Competing Determinants of Sample Types for SIA

SIA samples are generally made either of a particular tissue or of whole organisms. Single‐tissue samples, such as blood or muscle, are often preferred for analyses of *consumers* in *diet reconstructions* and for *comparing trophic positions and niches*, because their homogenous biochemical composition enables the comparison of SIA values between samples (Pinnegar and Polunin 1999). Moreover, each tissue provides dietary information over a specific temporal frame, which depends on the tissue metabolism and turnover rates, thus having similar temporal information from all samples (Vander Zanden et al. 2015). Preferred tissues typically have low fat and carbonate content, which would lead to depleted or enriched C isotopic signatures (e.g., molluscs' shell, crustaceans' exoskeleton, body fat; Post et al. 2007) and the associated interindividual or interspecific sample variability. By contrast, whole‐organism samples may be desired for *prey* in *diet reconstructions*, when entire animals are ingested by the predator. However, such samples integrate the isotopic composition of multiple tissues, each with different biochemical compositions, turnover rates (and thus, temporal information), fat or carbonate content, gut or glandular contents, and so forth (Mateo et al. 2008; Vander Zanden et al. 2015; Shipley and Matich 2020). Therefore, studies aiming both at comparing trophic positions and at reconstructing the diet of multiple species require careful attention to the sample types to obtain for each taxon.

Practically, the choice between single‐tissue and whole‐organism samples is often largely driven by constraints extrinsic to SIA theory, and may even conflict with the investigation requirements. A logistic constraint comes from the body size of the taxon of interest. On one hand, single‐tissue samples are often taken from larger organisms, for which it would be difficult to obtain a sample representative of the whole body anyway (McCutchan et al. 2003). Besides, non‐lethal sampling is now standard practice at least for vertebrates in many countries to support animal welfare in scientific research, especially for species of conservation interest, and generates alternative single‐tissue samples for SIA, such as scales, fins, feathers, or claws, whose isotopic values can be converted into muscle isotopic values using linear regressions (Bearhop et al. 2003; Jardine et al. 2011; Hette‐Tronquart et al. 2012). On the other hand, bulk analyses tend to be used for small organisms, for which it would be practically difficult (or even impossible) to dissect enough of a single tissue, the only alternative being sampling a single tissue from multiple specimens and then *pooling* them together to obtain enough material for the isotopic analyses (Maraun et al. 2023). This latter method, nonetheless, is not always feasible, depending on the species' availability and catchability. As a result of this ensemble of determinants, different sample types are used together, sometimes defying SIA assumptions. Identifying significant deviations from theoretical assumptions would help define acceptable practices in the rise of large‐scale stable isotope databases, and with increasing logistical and financial constraints for SIA studies in ecology.

### 2.1 Comparing Trophic Positions and Niches

In the case where both single‐tissue and whole‐organism samples are used, the stable isotope values of the sampled species could be incomparable. For example, in freshwater ecosystems, for crayfish only the abdominal muscle is usually sampled and analyzed, while small crustaceans (e.g., gammarids) are usually processed whole. The resulting gammarids' mixture of tissues, which also includes their carbonatic exoskeleton (which has a relevant biomass), will have stable isotope values that are hardly comparable with those of the muscle‐only samples of crayfish. In fact, the average values for both C and N stable isotopes will probably be affected by this mixing of tissues. In our example, in particular, the C stable isotope values of gammarids will be shifted to more positive values, due to the presence of inorganic C from the exoskeleton in the samples (Mateo et al. 2008; Fritsch et al. 2026). Similarly, the N stable isotope values will be enriched, due to the presence of excretory organs (Fritsch et al. 2026). As a result, the trophic position and isotopic niche of the crayfish and gammarids could be incomparable, and a higher sample variability could reasonably be expected in gammarids because whole‐body samples integrate multiple tissues with different isotopic compositions and variable proportions among individuals, in turn also inflating their niche width (Figure 1; but see Fritsch et al. 2026). Moreover, the bias caused by comparing crayfish muscle and bulk gammarids could greatly overestimate the overlap between the isotopic niches of the two species (Figure 1; Fritsch et al. 2026). A similar issue arises when the reference tissue, typically muscle, is substituted by alternative tissues sampled with non‐lethal methods. The correspondence between their isotopic values and those of the reference tissue is generally established in preliminary analyses based on linear regressions with *R* ^2^ > 0.8 (e.g. Jardine et al. 2011; Hette‐Tronquart et al. 2012; Bélouard et al. 2019). This substitution may change the interindividual variance captured by the converted stable isotope values, which may result in reduced or inflated niche sizes and overlaps.

**Figure 1:**
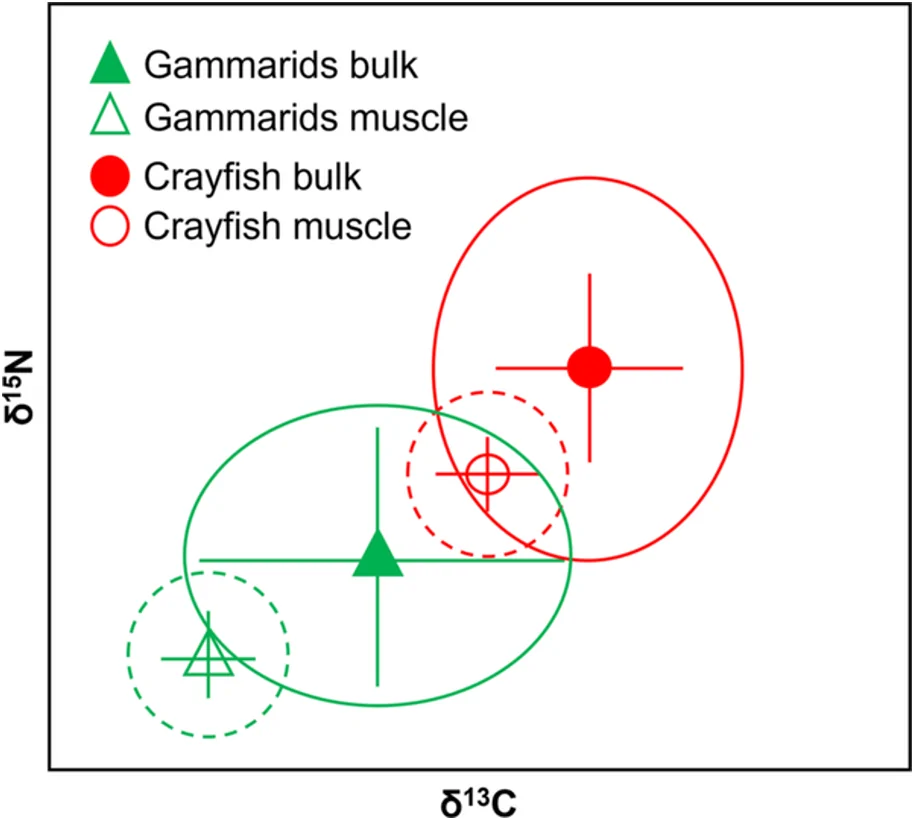
Conceptual illustration of the biases caused by comparing different sample types. Ellipses represent standard ellipses, and the symbols represent the average stable isotope values with associated error bars.

Using the same type of samples is thus important when comparing the position of species in the stable isotope space (e.g., their trophic position) and their niches (e.g., their niche breadth and overlap). Ad hoc experiments comparing trophic positions and niche breadth and overlap as estimated by SIA are needed to determine the extent to which these metrics are affected by the tissue type (Fritsch et al. 2026). Laboratory experiments comparing stable isotope values of whole‐body and specific tissues of animals reared in controlled conditions and fed with the same diet would provide such information. Alternatively, comparing subsamples of single tissues (e.g., muscle) with the rest of the animal from wild populations within the same individual could also be very informative, if the interindividual dietary variability could be considered negligible (e.g., in small ecosystems). Also, studies establishing linear equations between reference and alternative tissues should quantify the variance change in the conversion and how it affects niche metrics and niche overlap. These controlled comparisons could be extended across taxa, and informed by phylogenetic or biochemical similarities, offering broader generalizations.

Another promising method is represented by compound‐specific SIA (CS‐SIA), especially of amino acids. Indeed, essential amino acids tend to conserve their δ^13^C across trophic levels, while the δ^15^N of some (“source”) amino acids shows negligible TDFs, whereas other (“trophic”) amino acid show a strong enrichment (Chikaraishi et al. 2009; McMahon and McCarthy 2016; Whiteman et al. 2019). Interestingly, these patterns are relatively unaffected by the tissue type, with some exceptions (Matthews et al. 2024 and references therein). Thus, CS‐SIA could help make trophic positions and niches comparable where tissue heterogeneity is unavoidable. However, more studies and especially laboratory experiments with controlled diets comparing amino acids δ^13^C and δ^15^N among different tissues are needed to further support this approach and identify which trophic‐source amino acid pairs are less affected by tissue selection for trophic position estimates.

### 2.2 Diet Reconstruction

Using the same example, it appears clear that the estimated diets of crayfish and gammarids are incomparable, even if they feed on the same food sources, as the shift in the stable isotopes position of the bulk‐analyzed gammarids would bias mixing models results by altering the predicted link between prey and consumers (cf. the full vs. empty arrows representing the diet of gammarids in Figure 2a). Similarly, the proportion of crayfish and gammarids as prey in the diet of another consumer could also be biased by this shift in the stable isotope space (cf. the full vs. empty blue arrows in Figure 2a). This difference is even more accentuated if we consider that TDFs can vary among tissues (Stephens et al. 2023), and that the isotopic values of an organism analyzed whole depend on the mixture of multiple tissues and their proportion within the whole body.

**Figure 2:**
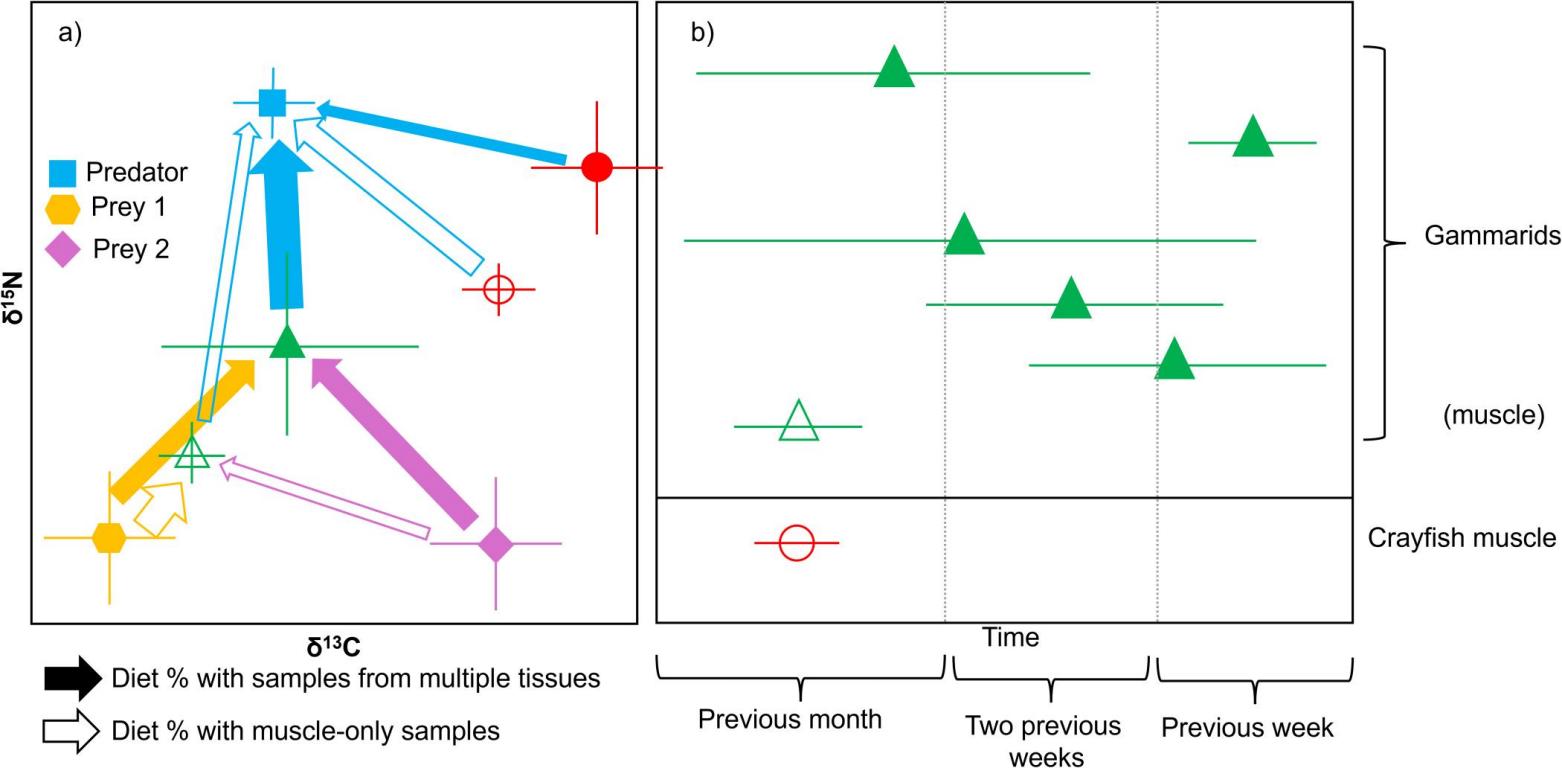
(a) Conceptual illustration of the biases caused by the use of different sample types in mixing models. The green triangles and the red circles have the same meaning as in Figure 1. The arrows indicate prey contribution to the diet of the consumer, and their thickness is proportional to their importance. (b) Conceptual illustration of the different temporal information provided by single‐tissue (open symbols) vs. whole‐body samples (filled symbols).

Additionally, the temporal window over which information on the diet is provided would differ between the two species, with isotopic values of crayfish muscle reflecting its diet over a specific period of time (e.g., the last month), while the time frame over which gammarids' diet has been reflected based on bulk samples is basically undeterminable (Figure 2b) and can vary greatly among individuals, according for example to the fat reserves, glandular content, gonad development, exoskeleton thickness, or gut fullness (this latter being probably the only thing that could be controlled for if animals are kept alive until they empty their digestive tract; e.g., Balzani et al. 2021).

Nonetheless, many consumers eat prey as a whole, and not just a specific tissue. Therefore, using just one tissue for the prey in diet reconstructions might lead to biased mixing models results (cf. the full vs empty blue arrows from gammarids in Figure 2a), since consumers' tissues are more likely to be derived from this global value than from a specific tissue. However, bulk samples are impractical for large prey and do not allow for a non‐lethal sampling, which is problematic even for some invertebrates (e.g., legally‐protected, or threatened species). Moreover, whole‐body analyses are more affected by potential contamination (e.g., residuals in the gut) and the inter‐individual variability in the proportion of each tissue, as discussed above. The two sexes could also differ considerably in the proportions of their tissues (e.g., between gonads and digestive apparatus).

Additional complexity arises from the fact that different tissues have different digestibility in the consumer's digestive apparatus, and their elements might contribute differently to tissue synthesis in the consumer (i.e., they can have a different isotopic routing; Boecklen et al. 2011). This complexity is further accentuated if we consider not only the multiple tissues of the prey but also the multiple tissues of the consumer, thus making this methodology both impractical and utterly complex.

Diet reconstruction based on multiple sample types thus requires technical developments, and thorough evaluation of candidate trade‐offs between accuracy and feasibility. A good compromise when analyzing the diet of a consumer is to analyze what the consumer actually eats, based on its ecology, and assimilates (whether whole organisms or just the soft tissues), with the consumer's samples being constituted of a single tissue that incorporates the signatures of these resources over the temporal window of interest. For example, crayfish are ingested whole by several fishes (e.g., catfish; Haubrock et al. 2018), while raccoons mainly eat their abdomen only (Boncompagni et al. 2021), making whole‐body analyses and abdominal muscles appropriate samples for the respective predators. However, since in trophic webs many species act as both consumers and prey, analyzing two samples, one for each trophic role for these “double players”, could represent a solution. In this way, the trophic web would be reconstructed through a series of finer‐scale predator‐prey mixing models. Also here, a comparison of stable isotopes mixing models from different tissues of experimental or wild (where reasonable) animals would provide an order of magnitude of this bias, and how much this varies across taxa.

Some of the complications explained above could be circumvented by CS‐SIA of amino acids. However, CS‐SIA also comes with its own hurdles. For example, gut microbes synthesize essential amino acids that the consumer can incorporate into biomass (Whiteman et al. 2018; Newsome et al. 2020), introducing another source of variation. Nonetheless, CS‐SIA seems a promising method to integrate SIA, especially for resolving complex food webs where tissue heterogeneity poses significant challenges.

### 2.3 Pooling Samples

Pooling individuals is a common practice, either to limit the number of samples to analyze, to reach the necessary material for SIA analyses, or when individual variability is not of interest. However, this reduces the inter‐individual variability that is usually taken into account with replicates, resulting in more precise values (i.e., lower variance). If we compare an individual‐based single‐tissue samples scenario, where both crayfish and gammarids have a certain mean and variance (Figure 3a), with a scenario where only gammarids were pooled using different single‐tissue samples, these latter will probably have a similar mean, but a lower variance (Figure 3b), since the variance between pools is likely lower than between individuals. Similarly, comparing an individual‐based whole‐body samples scenario, where both crayfish and gammarids have a shifted mean and larger variance (Figure 3c) with respect to the individual‐based single‐tissue samples scenario (Figure 3a), pooling whole‐body samples reduces variability between individuals, again probably resulting in a lower variance (Figure 3d). As a consequence, the trophic position of crayfish and gammarids would be still comparable, but not their niche width, which would be much more packed with pooled samples. Moreover, this variance reduction would result in differences in the diet estimation, both with gammarids as consumers and as prey, since mixing models specifically require a sample variability for consumers and a measure of variance (e.g., SD) for prey (Stock et al. 2018; Govan et al. 2023).

**Figure 3:**
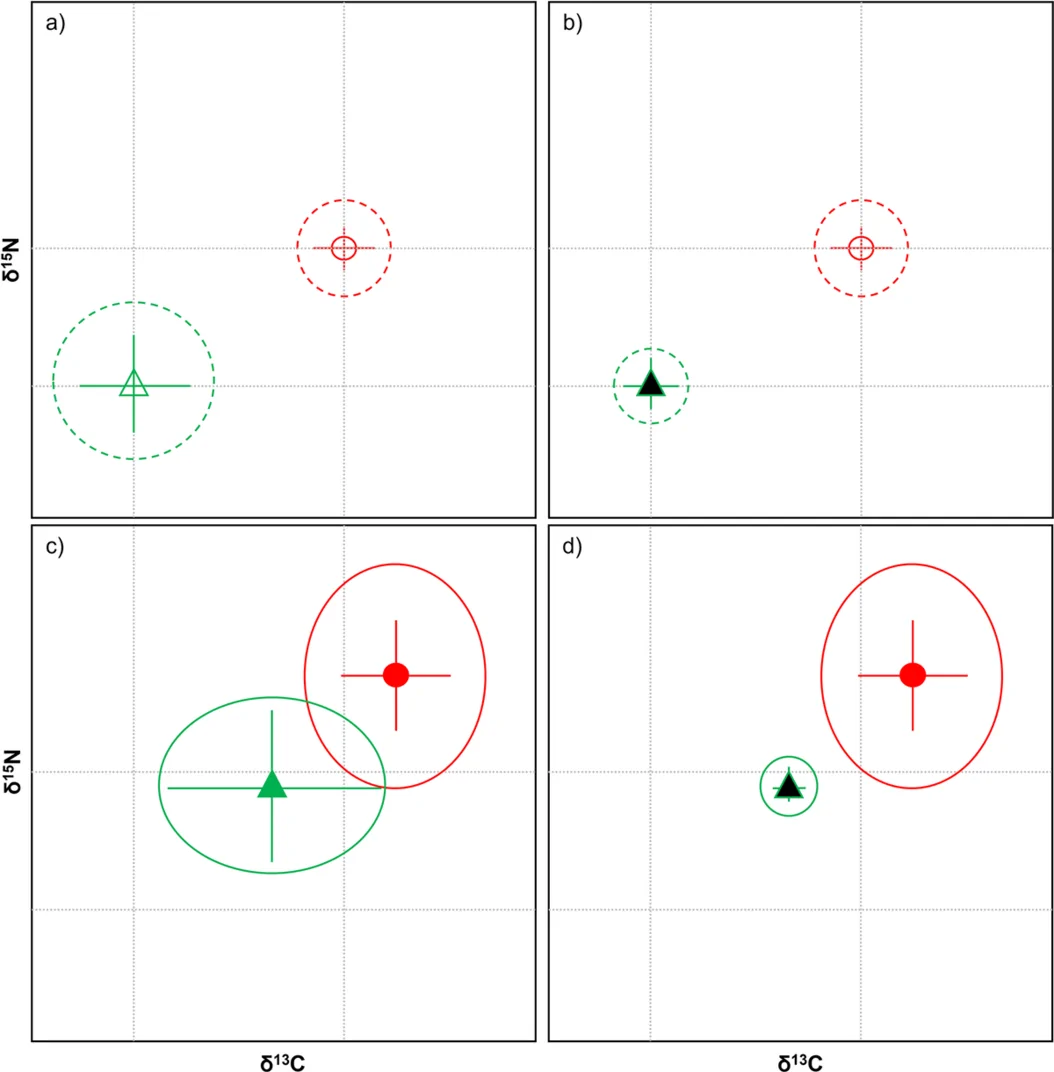
Conceptual illustration of the potential biases in comparing pooled and unpooled samples. Open symbols = single‐tissue samples; full symbols = whole‐body samples; black symbols = pooled samples. (a) scenario with individual‐based single‐tissue data; (b) scenario with single‐tissue data but where individuals of one taxon were pooled; (c) scenarion with individual‐based whole‐body data; (d) scenario with whole‐body data but where individuals of one taxon were pooled.

One potential solution for this situation could be to inflate the variance of pooled samples by making an assumption about the within‐pool variance. Targeted methodological experiments could however help quantify the effects of these differences. For example, small‐bodied organisms reared under controlled conditions and diet and dissected would allow comparisons of their isotope values among dissected tissues, whole‐body samples, both as single‐individual and pooled samples. Such efforts could support the development of correction factors or decision‐making frameworks to improve comparability across sample types.

### 2.4 A Call for Discussion

Overall, we call for a thoughtful consideration of the type of sample used for SIA, planning carefully the sampling design in order to answer specific ecological questions or hypotheses. All the potential limitations that we described may also apply within the same species, for example if adults and juveniles are analyzed using different sample types (e.g., Veselý et al. 2021). Our aim here was not only to highlight the uncertainties and potential biases underlying SIA (as well as the analyses of other biotracers following similar assumptions, e.g., fatty acid analysis), which are (one of) the elephant(s) in the room for ecologists comparing stable isotope values from different tissues for consumers. We proposed and called for ad hoc experiments to quantify the biases introduced by using multiple sample types in ecological SIA studies. Additionally, we specifically aimed to ignite a discussion among experts on how to implement a robust framework dealing with sampling considerations in a both pragmatic and theoretical way. This discussion would be beneficial especially in the light of the recent efforts to build specific FAIR repositories (e.g., Shipley et al. 2024; Boulêtreau et al. 2025), where the sample type is included as metadata, and that allow researchers to gather and compare stable isotope data from multiple studies spanning different years and countries.

## Author Contributions

P.B. and A.K. conceptualized the manuscript. P.B. wrote the original draft. N.B, L.V., and A.K. reviewed and edited the manuscript.

## Funding

The authors have nothing to report.

## Conflicts of Interest

The authors declare no conflicts of interest.

## Data Availability

The authors have nothing to report.
